# Analyzing the Effectiveness of AI-Generated Patient Education Materials: A Comparative Study of ChatGPT and Google Gemini

**DOI:** 10.7759/cureus.74398

**Published:** 2024-11-25

**Authors:** Nithin Karnan, Jobin Francis, Ishan Vijayvargiya, Christopher Rubino Tan

**Affiliations:** 1 Internal Medicine, K.A.P. Viswanatham Government Medical College, Tiruchirappalli, Tiruchirappalli, IND; 2 Emergency Medicine, Aneurin Bevan University Health Board, Newport, GBR; 3 Internal Medicine, Sir Seewoosagar Ramgoolam Medical College, University of Mauritius, Belle Rive, MUS; 4 Internal Medicine, Our Lady of Fatima University, Valenzuela, PHL

**Keywords:** artificial intelligence, chatgpt, claustrophobia, google gemini, magnetic resonance, mammography, mr, mri, mr safety, patient education brochure

## Abstract

Objective: The study aims to compare ChatGPT and Google Gemini-generated patient education guides regarding claustrophobia during MRI, mammography screening, and MR safe and unsafe items and the importance of knowing what items can be carried into an MR room.

Methods: The study utilized ChatGPT 3.5 and Google Gemini to create patient education guides concerning claustrophobia during MRI, mammography screening, and MR safe and unsafe items. A Flesch-Kincaid calculator was used to evaluate readability and ease of understanding. QuillBot (QuillBot, Inc., Chicago, USA) was used to generate a similarity score to evaluate possible plagiarism. In order to assess the scientific reliability of the AI-generated responses, we utilized a modified DISCERN score. R Studio 4.3.2 (The R Foundation for Statistical Computing, Vienna, Austria) was used for statistical analyses, with unpaired t-tests used to determine statistical significance between variables.

Results: The average number of words in ChatGPT and Google Gemini were 468.7±132.07 and 328.7±163.65, respectively. The mean number of sentences was 35.67±18.15 for ChatGPT and 30.33±12.22 for Google Gemini. Ease of readability for ChatGPT responses was 36.30±7.01 and for Google Gemini 46.77±4.96. The similarity scores for the ChatGPT responses were 0.50±0.62 and for Google Gemini 9.43±6.20. The reliability score was evaluated at 2.67±0.25 for ChatGPT and 2.67± 0.58 for Google Gemini.

Conclusion: The AI generated by ChatGPT and Google Gemini had no statistically significant difference in regard to word count, average word per sentence, average syllables per word, grade level comprehension score, or scientific reliability. However, the ease score was significantly greater for the ChatGPT response compared to Google Gemini. In addition, the similarity score was much higher in Google Gemini than in ChatGPT responses.

## Introduction

Magnetic resonance imaging (MRI) is a vital imaging technique in diagnostic radiology. However, the MRI process involves traveling through an imaging tunnel; patients are told to remain still and flat with the scanner ceiling hovering quite near their faces. Such factors have led to complaints of claustrophobia and a fear of enclosed space, and consequently, these fears may ultimately result in visual artifacts from patient motion or even patient cancellation of the procedure [1,2].

Therefore, patient education is a vital strategy to mitigate anxiety and enhance patient cooperation during MRI examinations. Traditionally, radiologists and other members of the healthcare team rely on verbal explanations to educate patients about standard MRI procedures, thus alleviating fears and behavior associated with claustrophobia during an MRI examination [1]. Recent advances and the proliferation of artificial intelligence (AI) have demonstrated potential in both diagnostic imaging and patient education [3,4]. The use of Al to create patient education guides may improve a patient’s familiarity with MRI studies, safety, and compliance, further promoting patient understanding and safety, while reducing patient anxiety.

Breast cancer (breast CA) remains a significant cause of mortality in women, with early mammography screening in susceptible populations being the most critical step in diagnosis and treatment [5]. Furthermore, patient education is the cornerstone of informed patient decision-making and the promotion of mammography screening. Notwithstanding the advantages of routine mammograms, patients may become confused and unwilling to participate if they are not informed about what a mammogram involves, why it is necessary for diagnosis, or how to interpret the results [6]. In addition, the promotion of mammography screening and informed patient decision-making depends heavily on patient education. Therefore, Al may be used to create educational materials for patients to increase participation and compliance.

Although MRI is a widely used diagnostic imaging modality that provides detailed images of internal organs and tissues without patient exposure to radiation, the unique environment of MRI poses potential risks, particularly concerning the presence of magnetic objects. Accidental exposure to magnetic materials in an MR suite may result in serious injuries or even fatalities, thus necessitating patient education regarding what are considered safe items in an MR suite [7].

Despite efforts to enhance safety protocols and minimize risks, patient awareness and understanding of what constitutes safe and unsafe items in the MRI room remain variable or, at worst, non-existent, resulting in preventable patient harm. Al can now be used to create patient education guidelines regarding safe versus unsafe items in the MRI environment, resulting in greater patient safety.

Aims and objectives

To compare ChatGPT and Google Gemini generated responses for writing patient education guides on claustrophobia during MRI, mammography screening, and safe and unsafe MR items and the importance of knowing what items can be carried to an MR room based on readability and ease of understanding. Additionally, the study aims to determine whether there are any significant differences in the grade level, ease score, similarity percentage, and reliability score of the patient education guides produced by AI chatbots.

## Materials and methods

This cross-sectional study took place over the course of one week, from April 1, 2024, to April 7, 2024. The present study was deemed exempt from ethics committee approval in view of the lack of human participant data.

Two AI chatbots, ChatGPT version 3.5 and Google Gemini, were used to create patient education guides. The directions covered three topics: how to deal with claustrophobia during an MRI, how to obtain a mammogram, and how to understand what is safe and risky in an MRI. The AI chatbots were prompted to write patient education guides on different topics, "Claustrophobia during MRI," "Mammography screening," and "MR safe and unsafe items & importance of knowing what items can be carried to an MR room." The responses were collected in Microsoft Word (Microsoft® Corp., Redmond, USA).

The generated answers were then evaluated using various tools; the Flesch-Kincaid Calculator was used to check the grade level, word count, sentence count, average number of words in a sentence, average number of sounds in a word, and word count. This was performed to determine readability and understandability [8]. The QuillBot plagiarism tool (QuillBot, Inc., Chicago, USA) was used to match with information that already exists in a database. The goal of this step was to ensure that the patient education guides had originality and integrity [9]. The modified DISCERN score was used to check the reliability of scientific information in the patient education guidelines. It consists of five questions that evaluate the reliability of health information. Each question was scored as either 0 or 1. A total score of 5 indicated high reliability, whereas a score of 0 indicated low reliability in this scoring system [10].

The data were exported to Microsoft Excel, and statistical analysis and visualization were performed using R version 4.3.2 (The R Foundation for Statistical Computing, Vienna, Austria). An unpaired t-test was used to determine any significant differences between the grade level, ease score, similarity percentage, and reliability score of the patient education guides made by ChatGPT version 3.5 and Google Gemini. Statistical significance was set at p<0.05. Reporting of results was done in accordance with the Strengthening the Reporting of Observational Studies in Epidemiology (STROBE) guidelines [11].

## Results

ChatGPT and Google Gemini were used to generate brochures on patient education for claustrophobia during MRI, mammography screening, and safe and unsafe MR items.

Table 1 presents the characteristics of the responses generated by ChatGPT and Google Gemini. There was no significant difference in the word count (p = 0.3157), sentence count (p = 0.6975), average word per sentence (p = 0.3092), average syllables per word (p = 0.2302), grade level (p = 0.174), similarity percentage (p = 0.1286), reliability score (p = 1.000) between ChatGPT and Google Gemini. However, the ease score was significantly better for the ChatGPT-generated responses than for Google Gemini (p = 0.1102). Based on the p-values obtained in Table 1, the current study does not have enough evidence to conclude the superiority of one AI tool over another.

**Table 1:** Characteristics of responses generated by ChatGPT and Google Gemini * Unpaired t-test; P-values <0.05 are considered statistically significant.

Variables	ChatGPT		Google Gemini		P-value*
	Mean	Standard Deviation	Mean	Standard Deviation	
Words	468.7	132.07	328.7	163.65	0.3157
Sentences	35.67	18.15	30.33	12.22	0.6975
Average Words Per Sentence	15.20	6.09	10.47	1.31	0.3092
Average Syllables Per Word	1.83	0.06	1.77	0.06	0.2302
Grade Level	12.0	2.31	9.33	0.81	0.174
Ease Score	36.30	7.01	46.77	4.96	0.1102
Similarity %	0.50	0.62	9.43	6.20	0.1286
Reliability Score	2.67	0.58	2.67	0.58	1.000

**Figure 1:**
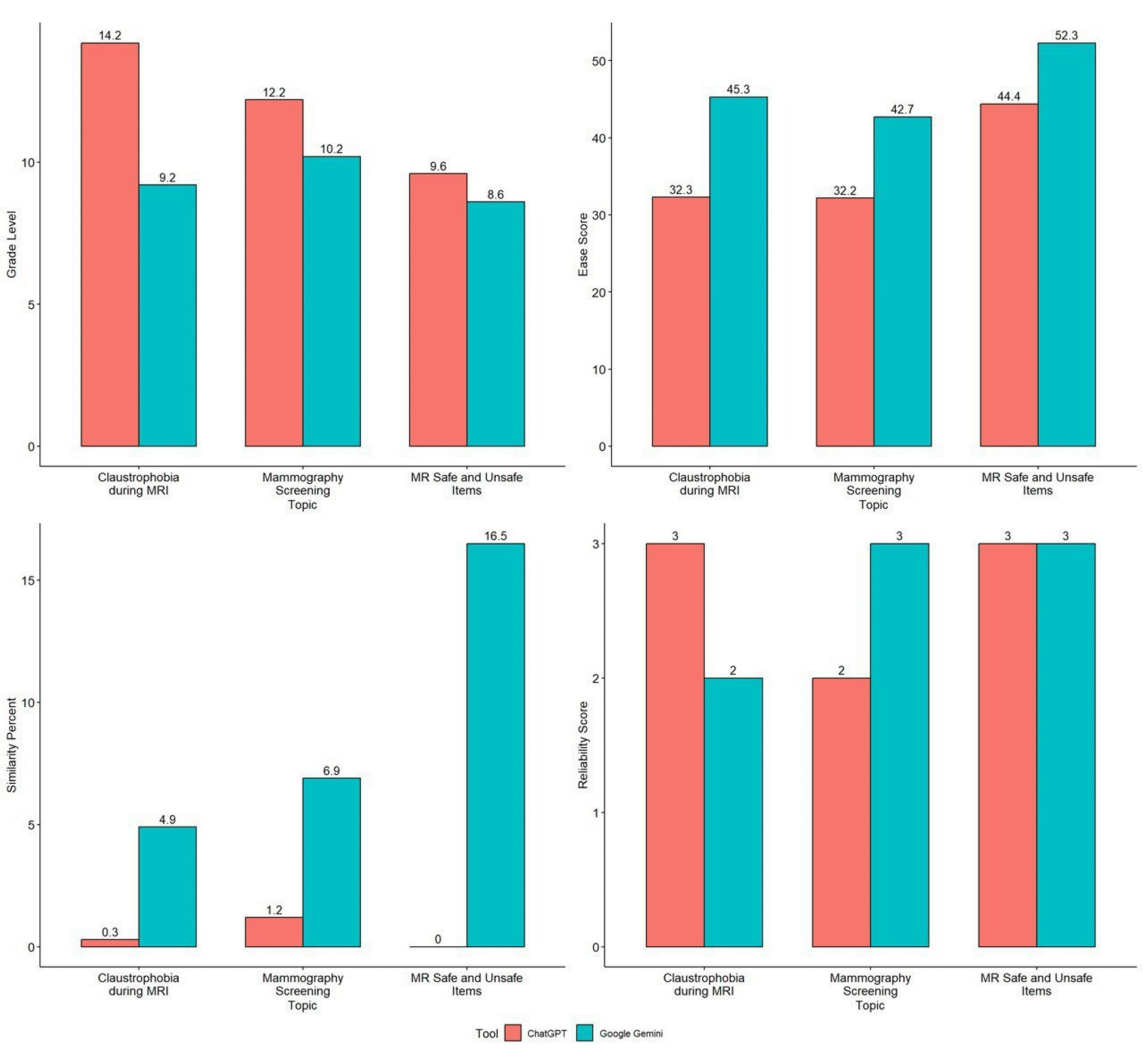
Comparison of the tools and the scores

Figure 1 shows a graphical representation of the comparison between the grade level, ease score, similarity percentage, and reliability score for the patient education guide generated by ChatGPT and Google Gemini. The ease score was significantly better for Google Gemini-generated responses (45.3, 42.7, and 52.3) than for ChatGPT (32.3, 32.2, and 44.4) for each brochure. The grade level is higher for ChatGPT (14.2, 12.2, 9.6) compared to Google Gemini (9.2, 10.2, 8.6). The similarity percentage was significantly higher for Google Gemini (4.9, 6.9, 16.5) than for ChatGPT (0.3, 1.2, and 0). The reliability score is 3 for ChatGPT response for "Claustrophobia during MRI" and "MR safe and unsafe items," whereas it is 2 and 3 for Google Gemini. The average reliability score for both AI-generated responses was 2.6.

## Discussion

This cross-sectional study was conducted to compare responses generated by two AI tools ChatGPT and Google Gemini for brochures on patient education for claustrophobia during MRI, mammography screening, and MR safe and unsafe items, revealed that that there is no significant difference in the average ease score, grade score and reliability score of responses generated by the two AI tools.

Advancements in AI have enhanced patient care and quality of life because the healthcare system is very intricate [11,12]. In one study, the quality and accuracy of the patient education brochure were evaluated using the Bing chatbot, a large language powered by ChatGPT for common radiological tests. The AI tool exhibited accurate responses for all inputs, indicating its potential for use in various aspects of patient education [13]. A research paper stated that online education platforms must have readability at the sixth to eighth grade level, but the majority of the platforms do not adhere to the guidelines because of various unresolved issues such as the extra time and cost needed to improve readability [14]. In the present study the ease score was 36.30 and 46.77 for ChatGPT and Google Gemini respectively, this shows that the readability of both tools is limited to people with higher education. According to another study, 89 responses were analyzed for average ease score, and it was concluded that the patient education brochure was of a higher grade than recommended, affecting the understanding of the general public [15].

In this study, there was no significant difference between the characteristics of both AI tools except for the ease score, which was significantly better in the responses generated by ChatGPT. Similar results were found in another study, in which 54 retinal detachment records entered into ChatGPT and Google Gemini were analyzed. Contingency analysis revealed significant differences between ChatGPT-4 and Gemini (p = 0.03). It was concluded that ChatGPT was better at generating accurate responses, which is similar to the findings of Carlà et al. [16].

AI tools have access to huge amounts of literature, so it is possible to produce responses similar to previously published articles, leading to an increase in ethical issues [17]. In the present study, the mean similarity percentage was 0.5 and 9.43 for ChatGPT and Google Gemini respectively. In another study, ChatGPT-4 was used to generate responses on fertility preservation in men and prepubescent boys, and plagiarism was found to be minimal [18].

The modified DISCERN score was used to assess the quality and authenticity of any study [19]. In this study, the mean modified 5-point DISCERN score was 2.67 for both AI tools. This implies that the reliability of the data is the same for both AI tools. In another study, the mean modified DISCERN score for AI-generated responses for ophthalmic abstracts using the two different versions of an AI tool were 35.9 and 38.1 (maximum of 50) for the earlier and updated versions, respectively (p = 0.30) [20]. In another study, the reliability and readability of three AI tools were compared, and ChatGPT was found to be superior to the other AI tools [21,22].

This study had several limitations; firstly, only two AI tools were compared, so there is a need to use various other available AI tools, as it will help in comparing different responses on a larger scale. Moreover, only three common radiological scenarios were analyzed, so this could make the study less applicable, and it is necessary to diversify the study to include numerous other conditions. The ChatGPT that was used in the study is an outdated version, so the responses generated might not be up-to-date. With progress made in the field of research, these tools must provide updated information.

## Conclusions

According to the study, there was no significant difference in the reliability score, average ease score, and grade score of responses generated by the two AI tools for patient information brochures on claustrophobia during MRI, mammography screening, and MR safe and unsafe items. There was no correlation between the reliability and ease scores of AI tools.

Several studies must be conducted to analyze the responses generated by different AI tools in common and more recent diseases. The ability of AI tools to generate the most recent information must be evaluated. These tools must provide the latest verified information to a larger section of the population.
